# Patients’ perspectives regarding hospital visits in the universal health coverage system of Thailand: a qualitative study

**DOI:** 10.1186/s12930-018-0046-x

**Published:** 2018-09-03

**Authors:** Apichai Wattanapisit, Udomsak Saengow

**Affiliations:** 1 School of Medicine, Walailak University, Thasala, Nakhon Si Thammarat, 80161 Thailand; 2 Center of Excellence in Health System and Medical Research, Walailak University, Thasala, Nakhon Si Thammarat, 80161 Thailand

**Keywords:** Health insurance, Hospital visit, Outpatient, Universal health coverage, Utilisation

## Abstract

**Background:**

A universal health coverage policy was implemented in Thailand in 2002 and led to an increase in accessibility to, and equity of, healthcare services. The Thai government and academics have focused on the large-scale aspects, including effectiveness and impacts, of universal health coverage over one decade. Here, we aimed to identify patients’ perspectives on hospital visits under universal health coverage.

**Methods:**

A qualitative study was carried out in four public hospitals in rural Thailand. We collected data through focus group discussions (FGDs) and in-depth interviews (IDIs). The semi-structured interview guide was designed to elicit perspectives on hospital visits among participants covered by the Universal Coverage Scheme, Social Security Scheme or Civil Servant Medical Benefit Scheme. Data were transcribed and analysed using a thematic approach.

**Results:**

Twenty-nine participants (mean age, 56.76 ± 16.65 years) participated in five FGDs and one IDI. The emerging themes and sub-themes were identified. Factors influencing decisions to visit hospitals were free healthcare services, perception of serious illness, the need for special tests, and continuity of care. Long waiting times were barriers to hospital visits. Employees, who could not leave their work during office hours, could not access some services such as health check-ups. From the viewpoint of participants, public hospitals provided quality and equitable healthcare services. Nevertheless, shared decision making for treatment plans was not common.

**Conclusions:**

The factors and barriers to utilisation of healthcare services provide exploratory data to understand the healthcare-seeking behaviours of patients. Perceptions towards free services under universal health coverage are positive, but participation in decision making is rare. Future studies should focus on finding ways to balance the needs and barriers to hospital visits and to introduce the concept of shared decision making to both doctors and patients.

## Background

Thailand achieved universal health coverage in 2002, therefore, all Thais are guaranteed access to healthcare services [1]. The Universal Coverage Scheme (UCS) covers 75% of the Thai population [1]. About 16% of the population, who are private-sector employees, are covered by the Social Security Scheme (SSS). Also, 9% of Thais, who are government employees; retirees; and dependants, are under the Civil Servant Medical Benefit Scheme (CSMBS) [1, 2].

As the largest proportion, one goal of the UCS is to ‘equally entitle all Thai citizens to quality healthcare according to their needs, regardless of their socioeconomic status’ [3]. Consequently, between 2003 and 2010, the number of hospital admissions increased from 0.094 to 0.116 admissions/member/year, and the number of outpatient visits grew from 2.45 to 3.22 visits/member/year [1].

According to universal health coverage, primary care or ambulatory care is provided across Thailand. A total of 10,347 public health centres and 992 outpatient departments (OPDs) of public hospitals belong to the Ministry of Public Health [4]. However, about 5% of public health centres have one or more doctor, and most of them are located in the capital city, Bangkok [4]. Most doctors in the public sector work in public hospitals rather than in public health centres. Patients can also seek outpatient services in the private sector (17,671 private clinics and 322 OPDs of private hospitals) [4]. It seems that patients have freedom to utilise healthcare facilities depending upon their predisposing factors (e.g., social structure and health beliefs), enabling resource (e.g., income levels and type of insurance) and need (e.g., chronic disease and disease severity) [5, 6].

After more than one decade of launching universal health coverage in Thailand, several studies published recently have focused on: (i) coverage, effectiveness, and economic evaluation of universal health coverage [7–11]; (ii) impacts of universal health coverage on other aspects of health and the health system [12–14]; and (iii) universal health coverage of specific health conditions (e.g., prevention of diabetes mellitus, and renal dialysis) [15, 16].

Studies focusing on the perspectives of patients on outpatient services and hospital visits in public health sectors in the context of universal health coverage are scarce. This is a gap in knowledge and an important research question. This study aimed to identify the perspectives of Thai patients on hospital visits using a qualitative method.

## Methods

### Ethical approval of the study protocol

The study protocol was approved by the Human Research Ethics Committee Walailak University (protocol number 030/2016). Participation in this study was voluntary and all participants provided written informed consent.

### Study design

This qualitative study was part of a larger project to investigate the views of patients and doctors with regard to hospital visits. The project was carried out between October 2016 and September 2017. The qualitative approach of this study was phenomenological [17].

Data were collected by focus group discussions (FGDs) and in-depth interviews (IDIs), if appropriate, and evaluated using interpretive thematic analyses. The number of FGDs and IDIs was dependent on data saturation. The Standard for Reporting Qualitative Research, which comprises 21 items, was used to ensure the transparency of the study [18].

### Context and participants

The study was conducted at four district hospitals in rural areas of Nakhon Si Thammarat province, Thailand. All hospitals included in our study provided outpatient and inpatient services. We did not conduct the study at the authors’ workplaces because we did not wish to bias relationships between doctors and patients or between researchers and participants.

We recruited participants by purposive sampling. OPD patients aged ≥ 18 years from the study sites were invited after their medical consultations to participate in the study. We invited 1–2 groups of participants each day and conducted FGDs or IDIs on the same day. The recruitment focused on all types of health insurance to maximise the variety of participants. It was not limited to any age. We did not equalise the presentation of gender. All participants were covered by UCS, SSS or CSMBS.

### Data collection

One author (AW), a family physician with training and experience in qualitative methods, conducted all FGDs and IDIs. FGDs were the main method of data collection. IDIs were conducted depending on the preferences of the participants. Each FGD consisted of 4–8 participants. The interviewer provided verbal information on all aspects of the study, and asked the permission of the participants to record the conversations using a digital audio recorder. Participants were requested to complete a short questionnaire (five items) regarding personal information. The interviewer conducted FGDs and IDIs following an interview guide (Table 1) in Central Thai language or Southern Thai dialect depending on the participants’ wishes. The authors developed the interview guide and revised the questions based on the comments and suggestions of the grant reviewers. One research assistant took notes during the data collection process. Each FGD or IDI took 45–90 min.

**Table 1:** Interview guide

Questions
How often do you visit the hospital?
Why do you visit the hospital?
Which factors influence your decision to visit the hospital?
Which symptoms or diseases make you visit a doctor at the outpatient department at the hospital?
What are your feelings towards and how satisfied are you about the quality of free healthcare services?
What are your feelings towards and how satisfied are you about the process of free healthcare services?
Do you have any shared decision making with the healthcare team?
What do you feel, in terms of equity and dignity, about using free healthcare services?

### Data analysis

The audio record files were transcribed verbatim. The two authors (AW and US—a physician and researcher with experience in qualitative methods) conducted the thematic analysis following the method of Braun and colleagues [19].

First, the two researchers read the transcripts independently to familiarise themselves with the data, and generated the initial codes. Next, the researchers separately searched for themes that were relevant to the research questions, and reviewed those themes to develop the initial thematic maps. The researchers worked together in defining and naming the final themes. We produced a report by writing up the themes and sub-themes with relevant quotations. We identified participants using the number of FGDs or IDIs and type of health insurance so as to maintain the confidentiality and anonymity of participants. The results, including the themes and quotations, were translated from Thai to English at the time of manuscript writing.

## Results

**Fig. 1:**
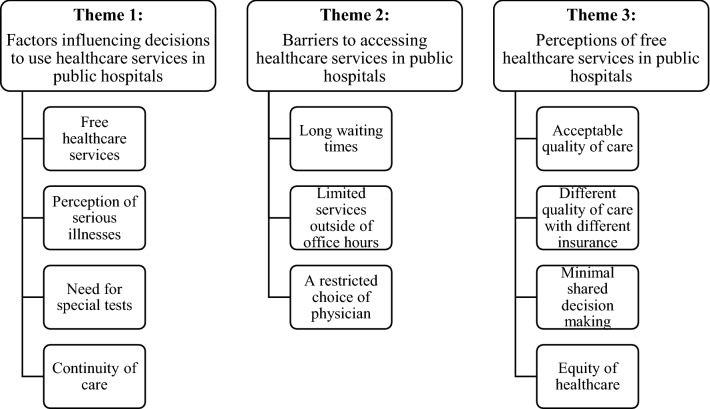
Summary of themes and sub-themes

We conducted five FGDs and one IDI between November 2016 and April 2017. Twenty-nine participants (mean age, 56.76 ± 16.65 years) participated in the study. Table 2 presents the characteristics of the participants. The findings consisted of three emerging themes: (i) factors influencing decisions to use healthcare services in public hospitals; (ii) barriers to accessing healthcare services in public hospitals; and (iii) perceptions of free healthcare services in public hospitals (Fig. 1).

**Table 2:** Characteristics of participants (n = 29)

Characteristic	n (%)
Age (years) mean (SD)	56.76 (16.65)
21–30	1 (3.4)
31–40	4 (13.8)
41–50	6 (20.7)
51–60	7 (24.1)
61–70	4 (13.8)
≥ 71	7 (24.1)
Sex	
Male	9 (31.0)
Female	20 (69.0)
Employment status	
Yes	19 (65.5)
No	10 (34.5)
Education	
Tertiary	6 (20.6)
Secondary	9 (31.0)
Primary or below	14 (48.3)
Income (Baht/month)^a^ mean (SD)	9160.34 (7667.99)
Health insurance	
UCS	12 (41.4)
SSS	7 (24.1)
CSMBS	10 (34.5)

Data presented as mean (SD) or n (%)

*UCS* Universal Coverage Scheme, *SSS* Social Security Scheme, *CSMBS* Civil Servant Medical Benefit Scheme, *SD* standard deviation

^a^35.79 Baht = US$1

### Theme 1: factors influencing decisions to use healthcare services in public hospitals

Self-care by seeking medications from nearby pharmacists was the first choice for treatment of non-severe illnesses. Patients, who needed to see doctors, could go to private clinics, private hospitals or public hospitals. According to data analysis, the facilitators to visits to public hospitals were as described in the sub-themes detailed below.

#### Free healthcare services

Compared with services in the private sector, use of healthcare services in public hospitals could save money for patients. Some participants sought diagnoses and initial treatments from private clinics or private hospitals. Later, they decided to receive free healthcare services, for continuity of treatment or medications, in public hospitals to save money.

‘…because I have the Social Security Scheme. It is my right and it is free.’

(FGD 3, SSS)

‘Prior to this visit, I went to a private clinic but I did not feel better after taking some medications. Next, I went to a private hospital and I got the diagnoses—hypertension and dyslipidaemia. I just needed to know the diagnosis and paid for the service. Today, I come here [a public hospital] because I think doctors are the same but I can get free medications here.’

(FGD 3, SSS)

#### Perception of serious illnesses

Visiting public hospitals was the first priority if serious or urgent conditions were presented. The decisions of participants were based on the belief that public hospitals had better capacities and facilities for such severe conditions compared with private clinics.

‘…severe conditions such as stomach pain—very painful. I will not go to a private clinic. Obviously, I have to go to the hospital. I have to stop drinking and eating here for pre-operative preparation if an operation is needed. The doctor at the private clinic does not do any major surgery and he would refer me to this hospital. So, I decide to visit the [public] hospital.’

(FGD 1, UCS)

#### Need for special tests

Participants, who needed blood tests or laboratory tests, preferred visiting public hospitals rather than seeking advice from private clinics. Past experiences with regard to special tests (e.g., a next-to-kin had a similar symptom and obtained the definitive diagnosis from blood tests) influenced their decision to visit hospitals.

‘I need a blood test to know the definitive diagnosis, so I come here [a public hospital]. If I went to a private clinic, they could not be able to take my blood for the test and they would refer me to the [public] hospital.’

(FGD 2, UCS)

#### Continuity of care

Patients with an underlying disease or chronic illness were more likely to visit the same hospital even though they had other illnesses that were not associated with the underlying disease. They believed that the hospital could provide informational continuity and suitable care.

‘If I get sick from any disease, I will go to the [public] hospital. They have my profile and can start my treatment immediately.’

(FGD 3, SSS)

### Theme 2: barriers to accessing healthcare services in public hospitals

Using healthcare services at private clinics or private hospitals required paying out-of-pocket. Nevertheless, participants sometimes went to the private sector due to the limitations of public hospitals.

#### Long waiting times

From the perspectives of patients, there was an imbalance between demand and supply. A hospital visit consumed a lot of time. Patients had to wait in a queue in the early morning before office hours, and waited for a long time to see a doctor. Sometimes, this wait took a full day.

‘Many people! Wait in the queue with one or two hundred people. The service, itself, is good but very slow. I spend a whole day in the hospital.’

(FGD 2, UCS)

#### Limited services outside of office hours

Some services, including health check-ups and specialist clinics, were provided only during office hours. It was inconvenient for people or patients’ carers who could not leave their work to visit hospitals. Therefore, some participants went to private clinics or private hospitals to seek healthcare services.

‘I used to go to the [public] hospital on Saturday for a PAP smear. It is available from Monday to Friday. As a teacher, it is not easy to leave my students and the service is not available at the weekend. So, I go to a private clinic because I am free in the evenings and can see a specialist. The payment is quite high and my insurance does not cover the cost from private clinics. Although I cannot claim that payment, I must pay. If I leave for a check-up and I am not ill, my boss will suspect me. Of course, I am concerned about my students.’

(FGD 4, CSMBS)

#### A restricted choice of physician

There were fewer opportunities to see a specific doctor at public hospitals. If patients wanted to see well-known doctors in their neighbourhoods, they had to go to the private clinics of those doctors.

‘I cannot choose a doctor here [a public hospital]. I can choose a doctor if I go to his private clinic’

(FGD 4, CSMBS)

### Theme 3: perceptions of free healthcare services in public hospitals

Quality of care among various types of health insurance was not different. Some participants mentioned that the quality of medications was different between the UCS and CSMBS. However, some participants argued that doctors made their decisions based on patients’ conditions rather than the types of health insurance available.

#### Acceptable quality of care

Compared with services in the private sector, the quality of medical advice proffered by doctors and treatments was similar.

‘My rights are not different from those of people who have the Civil Servant Medical Benefit Scheme. The doctor told me that medications are the same, and that there is no need to go to a private hospital. The standard of treatment is the same whether or not I pay. It happened to me.’

(FGD 5, UCS)

#### Different quality of care with different insurance

Some participants expressed that the coverage of UCS and SSS was different from CSMBS. For example, the payment for an admission in a private room was not covered by UCS.

‘Actually, we get a lot of things, but we have to pay for some items.’

(FGD 2, UCS)

#### Minimal shared decision making

Most participants were not familiar with the concept of shared decision making. Doctors made decisions regarding treatments for their patients. If patients disagreed with the doctors’ recommendations, they could discuss it with the doctors in advance.

‘I always follow the doctor’s recommendations. Whether the doctor says oral drugs or injection, I will do it.’

(FGD 2, UCS)

#### Equity of healthcare

Health insurance was considered to be a type of human right enabling access to healthcare and it was not a type of social class.

‘Free healthcare service! It is not a second-class service. All the things—treatments, services, and doctors—are normal. There is no discrimination.’

(IDI 1, SSS)

‘For me, I have a social security card. It is fair for workers.’

(FGD 3, SSS)

## Discussion

This study identified the perspectives of patients towards hospital visits. Free healthcare services, perception of serious illnesses, the need for special tests, and continuity of care were factors that influenced patients’ decisions with regard to visiting public hospitals. Barriers to visiting public hospitals included long waiting times, limited services outside of office hours and a restricted choice of physician in the public system. Free healthcare services were characterised as acceptable quality, paternalistic care, and equitable service.

We found that free healthcare services were one of the factors influencing the administration of public utilities. Studies in Thailand have supported the notion that universal health coverage increases healthcare utilisation in terms of hospital admissions and outpatient visits [14, 20]. This finding could reflect that patients, as consumers, may not be concerned about the cost associated with healthcare, which leads to the overuse of healthcare services [21]. A systematic review by Babitsch et al. [22] stated that perceived health status is one of the factors associated with use of healthcare services. This observation could explain our finding that people with serious or severe illnesses are more likely to visit hospitals. Patients’ expectations relating to test results could explain why they visit hospitals [23, 24]. We found that chronic illnesses are reasons for using healthcare services. This trend has also been found in different settings in Italy, China and Korea [6, 10, 25].

Long waiting times in OPDs were considered to be barriers for utilisation of healthcare services by study participants. Several studies in low-, middle- and high-income countries support the notion that long waiting times can cause stress and dissatisfaction in patients [26–28]. Similar to our study, research conducted in northern Nigeria found that the main reason for long waiting times was an imbalance in demand and supply (large numbers of patients with few healthcare workers) [27]. Accordingly, it seems that long waiting times in hospitals are common in developing countries [29]. Thailand has an 8-h working day, so public hospitals provide full services with maximum capacity of resources during office hours during the week. Conversely, they allocate some services with fewer resources outside of office hours. This scenario may be perceived as limited accessibility to the services required by employed people [2]. In contrast, the private sector has greater availability and more flexible services outside working hours and at weekends. Moreover, patients have less opportunities to choose their preferred doctors in the public system. This might lead to the discontinuity of care.

In Thailand, the coverage of health insurance has been expanded since UCS implementation in 2002 [30]. The UCS may lead to equity of healthcare among the Thai population, especially for those on low incomes, the unemployed, and people with chronic illnesses [2]. However, there is a need to deliver effective interventions to reach a higher standard of care, particularly for non-communicable diseases and long-term care [31]. Most importantly, the cost-effectiveness of the healthcare system should be considered based on the state of Thailand’s finances.

Our findings suggest that paternalistic healthcare is a common approach in a Thai context. Paternalism is thought to be able to control healthcare utilisation because doctors believe that he or she knows best, and makes decisions based on his or her views without the involvement of patients [32, 33]. A lack of shared decision-making between patients and doctors in clinical practice can be the effect of time constraints, patient characteristics, and clinical situations [34]. Whether increasing the participation of patients in decision making or shared decision making can reduce use and cost of unnecessary healthcare [35, 36], therefore, shared decision making might be an additional practice to improve care and reduce costs in the Thai healthcare system. We also asked the participants about their feeling in terms of dignity because patients are vulnerable and depend on the judgement, skill, and attitudes of the healthcare providers [37]. This may have an impact on the perspectives of patients regarding the healthcare services.

A main strength of this qualitative study was that it comprised participants with different characteristics, ages, and health insurance programmes which referred to universal health coverage. Moreover, it was conducted in several public hospitals. The different settings may be responsible for some variation in participants’ perceptions. A limitation of the study was that it was conducted in rural areas, so private hospitals in this context might not have been comparable with public hospitals, and the findings from a qualitative approach will not be generalisable to other populations, especially in urban settings. It is not known to what extent participants in this study represent rural hospital attendees in Thailand.

## Conclusions

The present study highlighted the perspectives of patients in rural areas in Thailand with regard to hospital visits. The factors and barriers to utilisation of healthcare services provide exploratory data to understand the healthcare-seeking behaviours of patients. Long waiting times in hospitals are due to an imbalance between the number of patients and providers, so effective management would entail resource optimisation. Services outside of office hours should be balanced between the requirements of patients and the resources available. In other words, enhancing the accessibility to healthcare must be realistic and cost-effective. A lack of choice to see a preferred doctor can interrupt the continuity of care. Perceptions towards free services in the context of universal health coverage were positive but participation in decision making was sparse. To improve the quality of healthcare services, there is a need to balance the needs and barriers to hospital visits, introduce the concept of shared decision making to healthcare providers, and reduce the inequity of access to healthcare.

## Abbreviations

CSMBS: Civil Servant Medical Benefit Scheme
OPD: outpatient department
SSS: Social Security Scheme
UCS: Universal Coverage Scheme
